# Comparison of the Inclination of Unerupted Mandibular Third Molars on Panoramic Radiography and Casts Made after Surgical Incision

**DOI:** 10.5681/joddd.2009.024

**Published:** 2009-09-16

**Authors:** Javad Yazdani, Farzad Esmaeili, Masume Johari

**Affiliations:** ^1^Assistant Professor, Department of Oral and Maxillofacial Surgery, Faculty of Dentistry, Tabriz University of Medical Sciences, Tabriz, Iran; ^2^Assistant Professor, Department of Oral and Maxillofacial Radiology, Faculty of Dentistry, Tabriz University of Medical Sciences, Tabriz, Iran

**Keywords:** Panoramic radiography, distortion, third molar

## Abstract

**Background and aims:**

Panoramic radiographs are used for surgical planning of unerupted third molars. The major prob-lems associated with panoramic radiography include unequal magnification and geometric distortion of the image. The purpose of this study was the clinical evaluation of the effect of radiographic distortion on the position and classification of unerpted mandibular third molars.

**Materials and methods:**

Panoramic radiographs of 20 patients with indication for extraction of lower third molars were included in this study. On the day of surgery, a silicon impression was taken from the second and third molar region and poured with type IV gypsum to provide a study cast. The inclination of the lower third molar to the second molar on panoramic radiog-raphy was compared with this angulation on the study casts.

**Results:**

There was a mean difference of 5.75° ± 1.65 between the position of the lower third molar on panoramic radiographs and on study casts. Student’s t-test indicated a statistically significant difference (P < 0.05).

**Conclusion:**

Panoramic radiography tends to exhibit a more mesial position of the third molars; however, panoramic radiog-raphy can still be used as the main tool for surgical planning of lower third molars.

## Introduction

Panoramic radiography is used for third molar surgical planning.^1^ However, this radiography provides a two-dimensional image and has its limitations. The main disadvantages of this technique include unequal magnification and distortion across the image, which may influence the interpretation of the image and finally the surgical planning of third molars.^2 , 3^

To the best of our knowledge, few articles have been published on distortion of panoramic images and its effect on the position of third molars. Therefore, the aim of the present study was to evaluate the effect of inherent distortion of panoramic radiography on the position and classification of unerupted mandibular third molars.

## Materials and Methods

In this cross-sectional study, 20 patients referring to the Faculty of Dentistry at Tabriz University of Medical Sciences, who were candidates for extraction of lower unerupted third molars, underwent panoramic radiography. The teeth with inverted position were excluded from the study. Informed consent was taken from all of the patients. The radiographs of all the patients were taken with a Planmeca Promax machine (Helsinki, Finland). All the radiographs were taken by an experienced operator and automatically developed.

**Figure 1:**
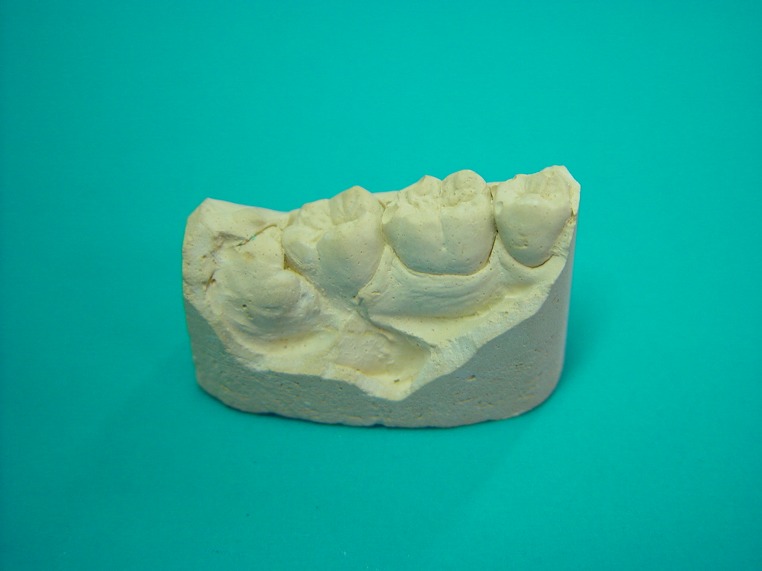
The cast made after surgical incision.

On the day of surgery, an impression was taken from the third and second molar region with additional silicone impression paste, which was disinfected with NaOCl spray, and rinsed with normal saline. After surgical incision and exposure of the crown of the third molar, the second impression was taken by silicone with good flow, which was disinfected and rinsed with water. Then the impression was poured with type IV gypsum to provide a study cast (Figure 1). The time interval between the radiographs and surgery did not exceed 1 month in any case.^4 , 5^

**Figure 2:**
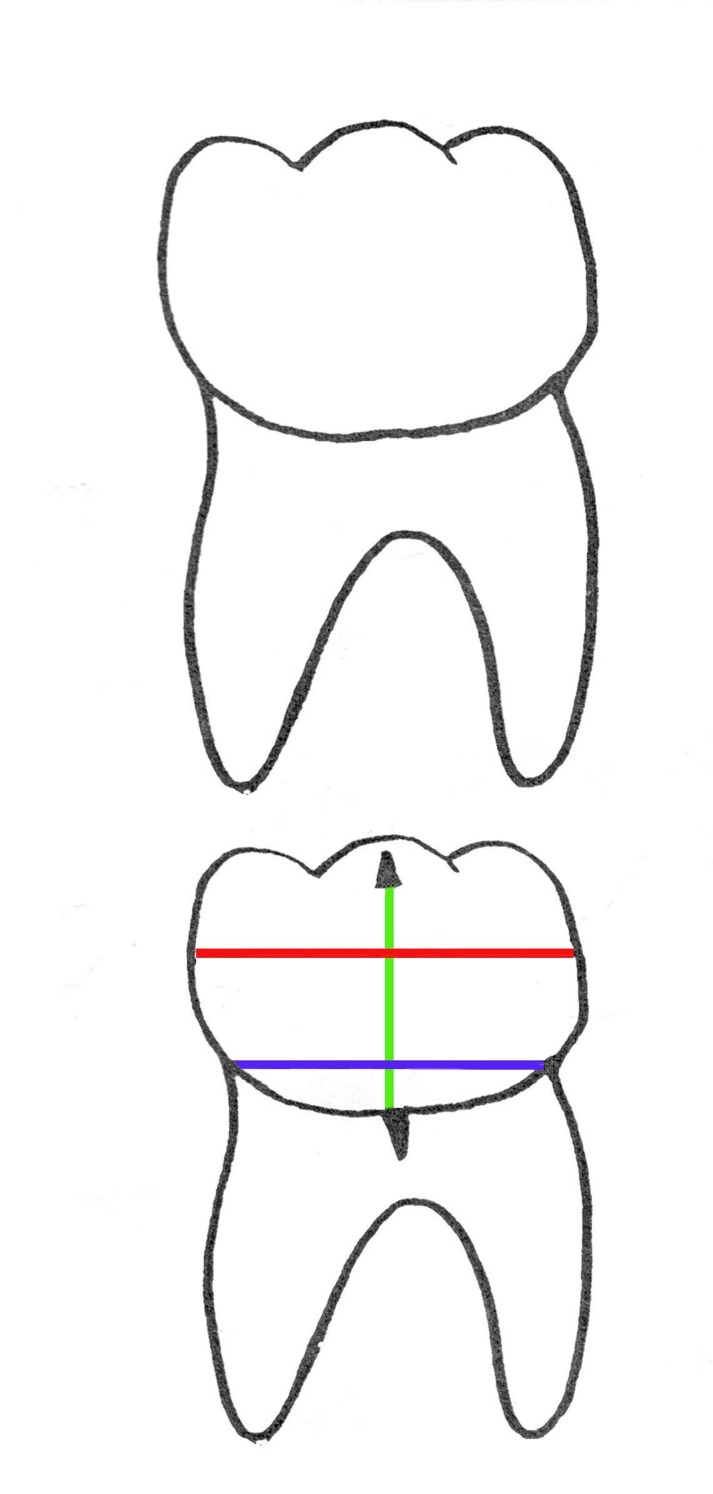
Method used for drawing the long axis of the teeth (refer to the text). The angulation between long axis of the second molar and third molar was used for classification of the third molars.

The relationship between the third and second molars on the traced panoramic radiographs was determined according to the three drawn lines. (1) through the cementoenamel junction in a mesiodistal direction; (2) through the point of greatest convexity of the crown in a mesiodistal direction; (3) through the midpoint of the other two lines (Figure 2), corresponding to the long axis of the tooth.^3^ Using a set square and a protractor, the inclination of the third molar to the second molar (angulation between long axis of these teeth) was measured. Then the third molars were classified as vertical, mesioangular, distoangular and horizontal (Table 1).^6^ These measurements were also made on the study casts and the teeth were classified. A comparison was made for the values of third molar angulation and classification between the study casts and panoramic radiographs. Descriptive statistics (for calculations of means and standard deviations of angulations) and student’s t-test for two paired samples were used.

**Table 1:** Angular values for classification of the mandibular third molars according to Winter 6

Angular values	classification
-30^ 0 ^ to -5^ 0 ^	Distoangular
-5^ 0 ^ to 5^ 0 ^	Vertical
5^ 0 ^ to 55^ 0 ^	Mesioangular
55^ 0 ^ to 105^ 0 ^	Horizontal

## Results

**Figure 3:**
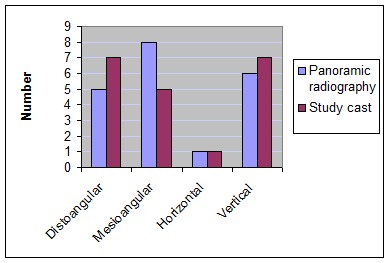
Frequency of position of mandibular third molars on panoramic radiographs and study casts.

From the 20 extracted third molars, one was radiographically classified as horizontal, eight had mesial angulation, six were vertical and five showed distal angulation. However, on the study casts, one was classified as horizontal, five had mesial angulation, seven were vertical and seven were classified as distoangular (Figure 3).

The mean degrees of inclination of the third molar to the second molar on panoramic radiographs and on study casts were 14.6^ 0 ^± 23.72 and 8.85^ 0 ^± 23.26, respectively. A mean variation of 5.75^ 0^ was observed in the angular measurements made on panoramic radiographs and study casts with a standard deviation of 1.65^ 0 ^. Student’s t-test for paired samples was used. This test showed that the measurements of the angles between the third and second molars on panoramic radiographs are different from study casts (P < 0.05).

## Discussion

Panoramic radiography is employed by most dentists for several purposes, especially for surgical planning of lower third molars in order to determine the inclination of the third molar to the second molar. The inclination of the lower third molar to the second molar is important for surgical planning. However, dentists should be aware of limitations and possible distortions of this technique.

According to our knowledge few articles have been published on the distortion of panoramic images and its effect on the position of third molars. Chandler et al ^7^ reported the precision of panoramic radiography for predicting the position of third molars and found a margin of error of 50% in the classification of teeth by dentists.

The present study clinically evaluated the effect of panoramic radiography distortion and demonstrated a mean alteration of approximately 5.5^ 0 ^, when measurements made on panoramic radiographs were compared with study casts, which means that panoramic radiography tends to exhibit a more mesial position for third molars, confirming the findings reported by Sant’Ana et al.^3^ In their study radiographs were taken by another machine; however, the results of the two studies are similar.

Therefore, during interpretation of the panoramic image, this fact should be considered that the teeth will actually be more distally positioned.

The results of this study coincide with the results of other studies on angular distortion of panoramic radiographs, which have reported distortion ranges from 5^ 0^ to 6^ 0 ^. ^8 , 9^

With regard to the frequency of third molar position on panoramic radiographs, higher frequency was related to the mesial angulation followed by the vertical position. However, study casts showed a higher frequency of teeth with distal angulation followed by the vertical position. Previous investigations have shown that mesial angulation is the most prevalent position of third molars.^10 , 11^These studies have evaluated the position of third molars radiographically. According to the results of the present study panoramic radiography tends to exhibit a more mesial position of the teeth andwe thought that most third molars might have distoangular positions. However, this assumption should be evaluated by other studies with larger sample sizes.

All the radiographs were taken by an experienced operator, which resulted in reduced errors and distortion of the technique.

In our study all the surgeries were performed within 1 month after taking the radiographs in order to ensure that the position of the teeth would not change during this period because some investigations have shown that the position of the unerupted third molar may change with time.^4 , 5^ Therefore, recent radiographs should be used for evaluation of third molar position.

In conclusion, panoramic radiography predicts third molar position more mesially. Other radiographs, such as periapical, should also be used for determination of third molar inclination and position. However, panoramic radiography can still be used as the main tool for surgical planning of third molars.
